# Association of Hyperuricemia with the Onset and Progression of Type 2 Diabetic Retinopathy

**DOI:** 10.3390/jcm15145573

**Published:** 2026-07-16

**Authors:** Akifumi Kushiyama, Iori Yamazaki, Haruka Ota, Momoka Kojima, Takako Kikuchi

**Affiliations:** 1 Department of Pharmacotherapy, Meiji Pharmaceutical University, Tokyo 204-8588, Japan; y191300@alm.my-pharm.ac.jp (I.Y.); y171058@alm.my-pharm.ac.jp (H.O.); y191118@alm.my-pharm.ac.jp (M.K.); tacky0720@gmail.com (T.K.); 2 Department of Diabetes and Metabolism, The Institute for Medical Science, Asahi Life Foundation, Tokyo 103-0002, Japan

**Keywords:** hyperuricemia, diabetic retinopathy onset, diabetic retinopathy progression

## Abstract

**Background/Objectives**: The relationship between hyperuricemia and diabetic retinopathy (DR) remains controversial despite both conditions being associated with vascular endothelial dysfunction. This study aimed to investigate the longitudinal association between hyperuricemia and the onset and progression of DR in patients with type 2 diabetes mellitus (T2D). **Methods:** We conducted a retrospective cohort study using patient data from initial visits between 2005 and 2022. Patients were categorized at baseline as having no DR (NDR), simple DR (SDR). The primary endpoint was the onset of DR from NDR, and the secondary endpoint was the progression of DR from SDR. Kaplan–Meier and Cox proportional hazards analyses were employed for statistical evaluation. **Results:** The study included 1972 patients (1702 with NDR and 270 SDR). In the overall NDR cohort, hyperuricemia was not significantly associated with the onset or progression of DR. However, upon stratification by baseline HbA1c, hyperuricemia was significantly associated with a higher incidence of DR within the high-HbA1c subgroup (≥9%) of the NDR cohort (HR 2.40, 95% CI [1.27–4.55]). Similarly, within the SDR cohort, a Kaplan–Meier analysis demonstrated a significantly higher rate of DR progression in the hyperuricemia group, exclusively among patients with HbA1c ≥ 9% (*p* = 0.01). **Conclusions:** Hyperuricemia is independently associated with the onset and progression of DR in T2D patients with poor glycemic control While often co-occurring with other DR risk factors, hyperuricemia may serve as a potential marker for the development of retinopathy in this high-risk population.

## 1. Introduction

Diabetic retinopathy (DR), a hallmark vascular complication of diabetes mellitus, is a leading cause of acquired blindness globally [1]. Chronic hyperglycemia drives vascular endothelial dysfunction, a key pathological mechanism that cascades into neuropathy, retinopathy, and nephropathy [2,3,4]. Because of its insidious progression, DR can cause irreversible visual impairment before the manifestation of clinical symptoms. Clinically, DR is staged using the modified Davis classification into no DR (NDR), simple DR (SDR), pre-proliferative DR (PPDR), and proliferative DR (PDR).

Hyperuricemia, defined as serum uric acid (SUA) levels ≥ 7.0 mg/dL [5], frequently co-occurs with diabetes. In Japan, the 2022 National Health and Nutrition Survey by the Ministry of Health, Labour and Welfare reported hyperuricemia in approximately 20% of adult males and <5% of adult females [6]. The comorbidity of hyperuricemia and diabetes is tightly intertwined; hyperuricemia-induced endothelial dysfunction and nitric oxide inhibition potentially exacerbate insulin resistance [7], compounded by shared lifestyle factors such as a Westernized diet [8]. Mechanistically, uric acid (UA) promotes cellular oxidative stress via reactive oxygen species, disrupting the homeostatic balance between endothelin and nitric oxide, thereby aggravating vascular dysfunction [9,10,11].

Although the pathological link between hyperuricemia and diabetic nephropathy has been extensively investigated [12,13], the relationship between hyperuricemia and DR remains poorly elucidated [7]. Emerging evidence suggests that hyperuricemia may promote angiogenesis and chronic inflammation in DR, as indicated by elevated levels of vascular endothelial growth factor (VEGF) and high-sensitivity C-reactive protein (hs-CRP) levels [14]. Furthermore, cross-sectional analyses have also demonstrated that elevated SUA levels are independently correlated with advanced stages of DR [15,16].

While a prior longitudinal study indicated an increased DR risk in men with lower thresholds (4.79 mg/dL) of SUA levels [17], the clinical significance of the diagnostic threshold for hyperuricemia (≥7.0 mg/dL) remains unresolved under distinct metabolic conditions. Marked hyperglycemia induces glucosuria, wherein high concentrations of urinary glucose compete with urate at glucose transporter 9 (GLUT9) in the renal tubules, thereby enhancing renal UA excretion and typically lowering SUA levels [18]. This interplay suggests that the relationship between hyperuricemia and DR complications may be fundamentally modulated by a patient’s glycemic status. However, robust longitudinal evidence is lacking regarding whether clinical hyperuricemia serves as a reliable marker for DR onset or subsequent progression from SDR, particularly across varying degrees of glycemic control. To address these gaps, this study aimed to longitudinally investigate the association between hyperuricemia and the onset and progression of DR across different strata of glycemic control in patients with type 2 diabetes (T2D), thereby evaluating its utility as a clinical prognostic marker.

## 2. Materials and Methods

### 2.1. Study Design and Subjects

This retrospective cohort study utilized a patient database established through a joint research project involving the Department of Pharmacotherapy and the Department of Public Health and Epidemiology at Meiji Pharmaceutical University and the Institute for Medical Sciences, Asahi Life Foundation, comprising data from initial visits between 2005 and 2022. The study was conducted in accordance with the Declaration of Helsinki and approved by the Ethics Committee of the Institute for Medical Science, Asahi Life Foundation, and Meiji Pharmaceutical University (Approval No.: 14003 and 202435). The requirement for informed consent was waived due to the retrospective study design, and an opt-out consent procedure was adopted.

### 2.2. Definitions

(1) Baseline Data: Defined as the clinical and laboratory data obtained on the date closest to the initial fundus examination within a window of ±90 days;
(2) Assessment and Classification of DR: Fundus examinations were performed by ophthalmologists following the administration of appropriate mydriatics. The stage of DR was determined and documented in the ophthalmology-specific medical records. For this study, DR staging was retrospectively collected based on the ophthalmologists’ assessments and classified according to the modified Davis classification: NDR, SDR, PPDR, and PDR;
(3) Hyperuricemia: Defined as an SUA level of ≥7.0 mg/dL or 416 μmol/L. In accordance with the Japanese guidelines for the management of hyperuricemia and gout [8], which define hyperuricemia as a serum uric acid concentration ≥ 7.0 mg/dL, irrespective of sex, we applied this clinical criterion in the current study;
(4) HbA1c Stratification: Patients were categorized into three groups by HbA1c levels: <7%, 7% to <9%, and ≥9%.

### 2.3. Inclusion and Exclusion Criteria

The inclusion criteria were as follows: (1) a diagnosis of T2D and (2) at least two recorded fundus examinations. The exclusion criteria were as follows: (1) diagnosis of any other type of diabetes other than T2D, (2) PPDR or PDR at the initial fundus examination, and (3) lack of SUA or HbA1c measurements within the 90-day baseline window. Patients were stratified into two analytical cohorts based on their initial DR status: the NDR and SDR cohorts.

### 2.4. Primary and Secondary Endpoints

Primary Endpoint: Onset of any stage of DR from NDR.

Secondary Endpoint: Progression of DR from SDR by at least one stage.

### 2.5. Follow-Up and Censoring

The observation period commenced 90 days after the initial fundus examinations. For patients who did not experience the onset or progression of DR during the observation period, follow-up was censored at the date of their last recorded fundus examination.

### 2.6. Statistical Analysis

Baseline characteristics were summarized as number (N) (%), mean ± standard deviation (SD), or median (interquartile range, IQR). Group comparisons were performed using the chi-square test for categorical variables and an ANOVA for continuous variables. The associations between hyperuricemia and DR endpoints were evaluated using a Kaplan–Meier survival analysis with the log-rank test and Cox proportional hazards models.

To investigate the continuous dose–response relationship between SUA levels and the risk of retinopathy, a restricted cubic spline (RCS) analysis based on the Cox proportional hazards model was conducted. In the RCS modeling, four knots were utilized, and the reference hazard ratio was set to 1.0 at an SUA level of 7.0 mg/dL.

Multivariable Cox models were adjusted for age, sex, duration of diabetes, BMI, blood pressure, HbA1c, SUA, creatinine, and use of antidiabetic or UA-lowering medications. To balance these baseline covariates between hyperuricemia and non-hyperuricemia groups within each HbA1c stratum, propensity score matching (1:1 nearest neighbor, caliper = 0.2) was executed using the same clinical covariates, with the exception of SUA. Covariate balance post-matching was assessed using the standardized mean difference (SMD). In the multivariable analyses and propensity score matching, patients with missing data for any of the included covariates were excluded from the respective analyses (complete case analysis).

The primary analyses, including propensity score matching and Cox regression, were conducted using JMP Student Edition (ver. 19, SAS Institute Inc., Cary, NC, USA), while the RCS curves were generated using EZR (ver. 1.70, Saitama Medical Center, Jichi Medical University, Saitama, Japan), a graphical user interface for R (The R Foundation for Statistical Computing, Vienna, Austria). The results of the Kaplan–Meier and subgroup analyses were visualized using GraphPad Prism (ver. 11.0.2, GraphPad Software, Boston, MA, USA). *p*-values < 0.05 were considered statistically significant.

## 3. Results

### 3.1. Study Participants

**Figure 1:**
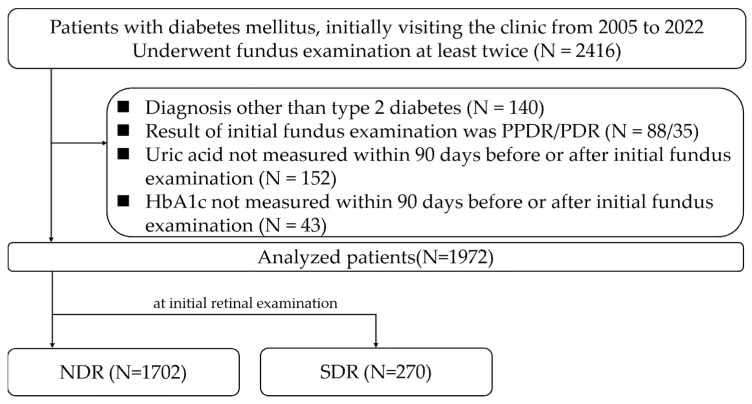
Flowchart of study participants.

Of the 2416 patients with at least two fundus examinations, 1972 met the inclusion criteria (Figure 1). The final cohort consisted of 1702 patients with NDR and 270 with SDR at baseline. The mean age was 55.1 ± 10.9 years, and 79.6% were male.

### 3.2. Baseline Characteristics

Baseline clinical characteristics stratified by hyperuricemia status are presented in Table 1 and Table 2.

In the NDR cohort (Table 1), patients with hyperuricemia (≥7 mg/dL or 416 μmol/L), compared to those without, were significantly more likely to be male, younger, and have a higher BMI, higher blood pressure, and lower eGFR. Paradoxically, the hyperuricemia group had a slightly lower mean HbA1c level. As expected, the utilization of UA-lowering medications was significantly higher in the hyperuricemia group. These clinical profiles were largely consistent within the NDR (Table 1) and SDR (Table 2) groups. In both groups, hyperuricemia was coupled with elevated BMI and compromised renal function.

### 3.3. Hyperuricemia and DR Onset (NDR Cohort)

**Figure 2:**
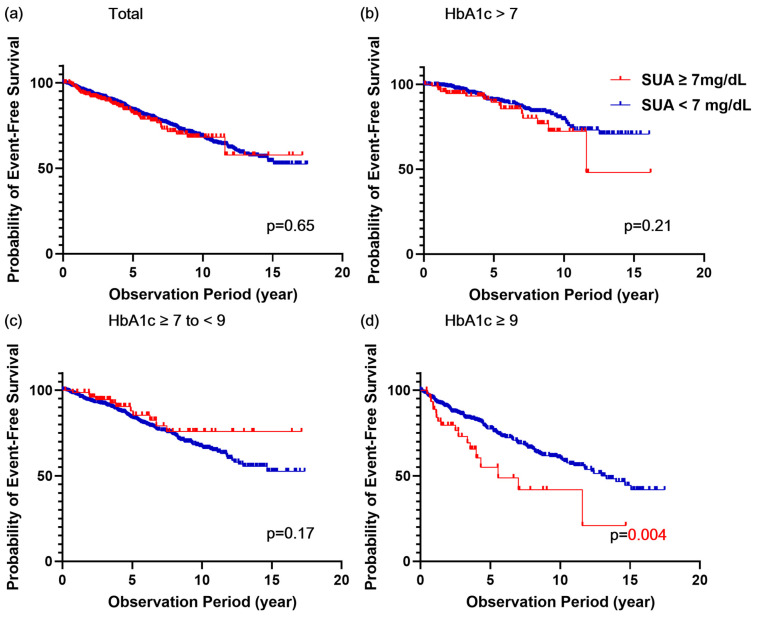
Kaplan–Meier survival analysis for DR onset in the NDR cohort. Event-free survival from the onset of DR stratified by hyperuricemia status. (**a**) All patients. (**b**) Patients with HbA1c < 7%. (**c**) Patients with HbA1c 7% to <9%. (**d**) Patients with HbA1c ≥ 9%. An SUA level of 7 mg/dL is equivalent to 416 μmol/L.

In the overall NDR cohort, the DR incidence rate was 37.3 per 1000 person-years in patients with an SUA level < 7 mg/dL (416 μmol/L), and 39.3 per 1000 person-years in those with a level ≥ 7 mg/dL (416 μmol/L). When stratified by HbA1c levels, in patients with HbA1c < 7%, the incidence rate was 21.6 per 1000 person-years for <7 mg/dL and 29.5 per 1000 person-years for ≥7 mg/dL (416 μmol/L). In patients with HbA1c 7 to 9%, the rates were 38.2 and 24.6 per 1000 person-years, respectively. Notably, in the highest glycemic category of HbA1c ≥ 9%, the incidence rate was 50.9 per 1000 person-years for <7 mg/dL (416 μmol/L), whereas it reached 109.5 per 1000 person-years for ≥7 mg/dL (416 μmol/L). The Kaplan–Meier analysis showed no significant difference in the time to the onset of DR between patients with and without hyperuricemia (Log-rank *p* = 0.65) (Figure 2a). However, upon stratification by baseline HbA1c, a significant association emerged. While no difference was observed in patients with HbA1c < 7% (Figure 2b) or 7% to 9% (Figure 2c), hyperuricemia was associated with a significantly shorter time to DR onset in patients with HbA1c ≥ 9% (Log-rank *p* = 0.004) (Figure 2d).

**Figure 3:**
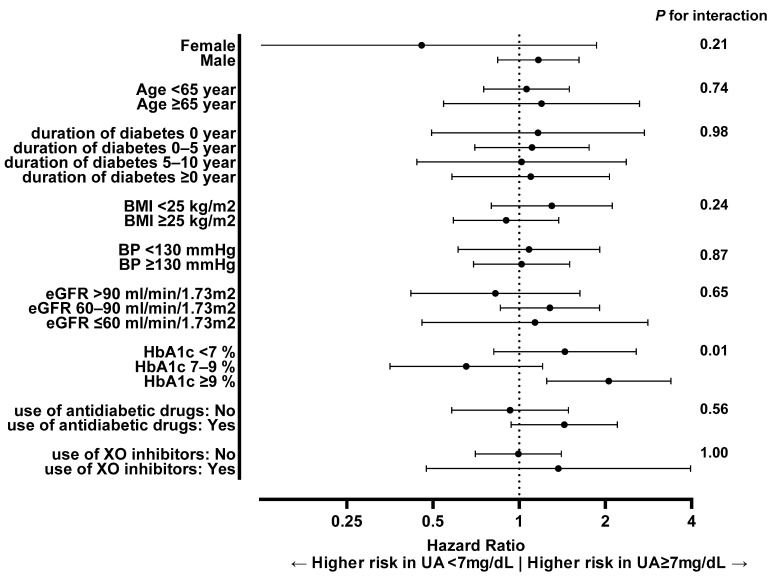
Subgroup analysis of the association between hyperuricemia and the incidence of retinopathy in the NDR cohort. Forest plot displaying the hazard ratios (black circles) and 95% confidence intervals (horizontal lines) for retinopathy development in the SUA ≥ 7 mg/dL (416 μmol/L) group versus the SUA < 7 mg/dL group. The data were stratified by key demographic and clinical variables. The *p*-values for the interaction are shown in the right column.

Furthermore, a multivariable Cox proportional hazards regression analysis revealed that HbA1c was the only clinical variable that exhibited a significant interaction with hyperuricemia regarding the onset of DR (*p* for interaction = 0.01; Figure 3). Consistent with the results of the stratified Kaplan–Meier analysis, the risk of DR onset associated with hyperuricemia was most pronounced in the highest glycemic category; among patients with a baseline HbA1c ≥ 9%, hyperuricemia was associated with a more than two-fold increased incidence of DR onset compared to non-hyperuricemia, with a hazard ratio of 2.05 (95% CI, 1.25–3.38). In contrast, no significant interactions or markedly elevated hazard ratios were observed in other subgroups (Figure 3).

**Figure 4:**
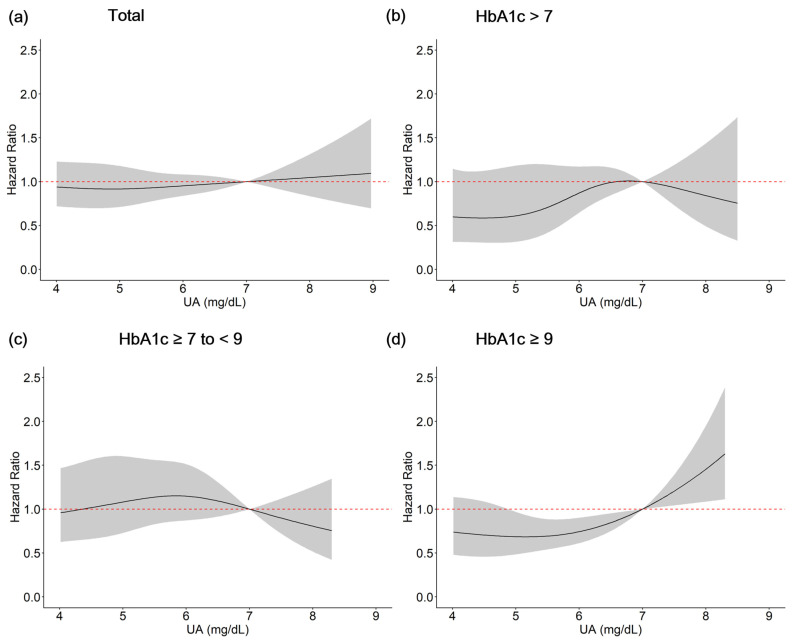
Dose–response relationship between SUA levels and the risk of diabetic retinopathy onset. Restricted cubic spline curves display the hazard ratios (solid black lines) and 95% confidence intervals (shaded areas) for the onset of diabetic retinopathy. The reference value for SUA was set at 7.0 mg/dL (416 μmol/L), indicated by the intersection with the horizontal red dashed line at a hazard ratio of 1.0. All hazard ratios are presented relative to this reference concentration. The analysis was conducted for (**a**) the total cohort, and stratified by baseline HbA1c levels: (**b**) HbA1c < 7%, (**c**) HbA1c 7% to <9%, and (**d**) HbA1c ≥ 9%.

To further investigate the continuous dose–response relationship between SUA levels and the incidence of DR, restricted cubic spline (RCS) curves were generated using a reference SUA level of 7.0 mg/dL (Figure 4). In overall cohort, the RCS model did not demonstrate a clear overall dose–response association (Figure 4a). However, consistent with the interaction analysis, distinct patterns emerged upon stratification by baseline HbA1c levels. In patients with HbA1c < 7% (Figure 4b) and those with HbA1c 7% to <9% (Figure 4c), elevated SUA levels above 7.0 mg/dL did not correspond to an increased risk of DR. Conversely, within the highest glycemic category (HbA1c ≥ 9%), the HR of DR onset exhibited a sharp, continuous increase as SUA levels rose above the 7.0 mg/dL reference point (Figure 4d). Notably, within this severe hyperglycemia subgroup, SUA levels in the range of approximately 5.0–7.0 mg/dL were associated with hazard ratios < 1.0 relative to the reference value (Figure 4d). This continuous modeling visually corroborates that while the statistical association between hyperuricemia and DR onset is highly pronounced under severe hyperglycemia, maintaining physiological SUA levels may offer a protective effect even within this high-risk stratum.

### 3.4. Hyperuricemia and DR Progression (SDR Cohort)

**Figure 5:**
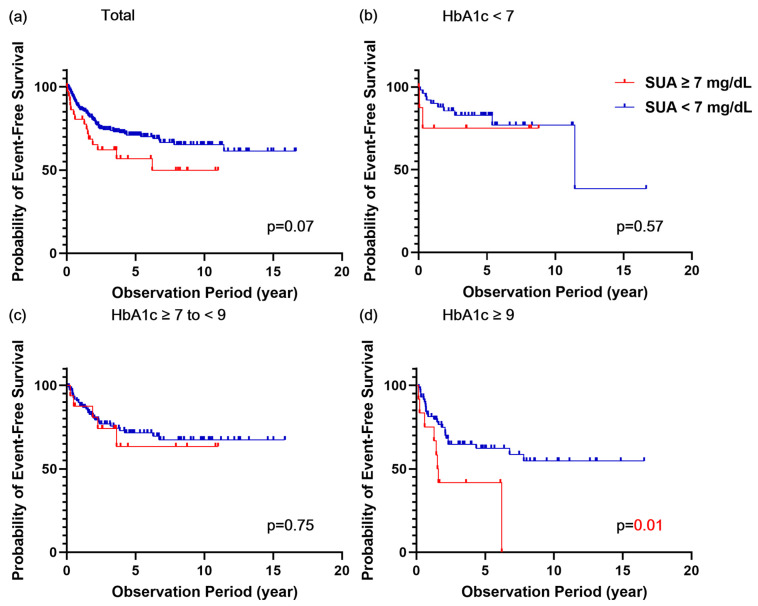
Kaplan–Meier survival analysis for DR progression in the SDR cohort. Event-free survival from DR progression stratified by hyperuricemia status. (**a**) All patients. (**b**) Patients with HbA1c < 7%. (**c**) Patients with HbA1c 7% to <9%. (**d**) Patients with HbA1c ≥ 9%. A serum urate level of 7 mg/dL is equivalent to 416 μmol/L.

In the SDR cohort, a parallel stratified analysis was performed for the SDR cohort. In the overall cohort, the incidence rate was 61.2 per 1000 person-years in patients with an SUA level < 7 mg/dL (416 μmol/L), and 117.5 per 1000 person-years in those with a level ≥ 7 mg/dL (416 μmol/L). When stratified by HbA1c levels, among patients with HbA1c < 7%, the incidence rate was 46.2 per 1000 person-years for <7 mg/dL (416 μmol/L) and 59.6 per 1000 person-years for ≥7 mg/dL (416 μmol/L). In patients with HbA1c ≥ 7%, the rates were 53.4 and 73.4 per 1000 person-years, respectively. Finally, in the highest glycemic category of HbA1c > 9%, the incidence rate was 85.9 per 1000 person-years for <7 mg/dL (416 μmol/L) and 307.9 per 1000 person-years for ≥7 mg/dL (416 μmol/L). The Kaplan–Meier survival analysis showed that patients with hyperuricemia overall tended to have a higher rate of DR progression, although this difference did not reach statistical significance (log-rank *p* = 0.07; Figure 5a). However, when stratified into three groups based on HbA1c levels at the initial retinal examination (Figure 5b–d) in the same manner as the NDR cohort, a significantly higher rate of retinopathy progression was observed in the hyperuricemia group exclusively among patients with HbA1c ≥ 9% (*p* = 0.01). Nevertheless, it should be noted that the sample size for each group with both HbA1c ≥ 9% was small (n = 35) after matching propensity scores.

**Figure 6:**
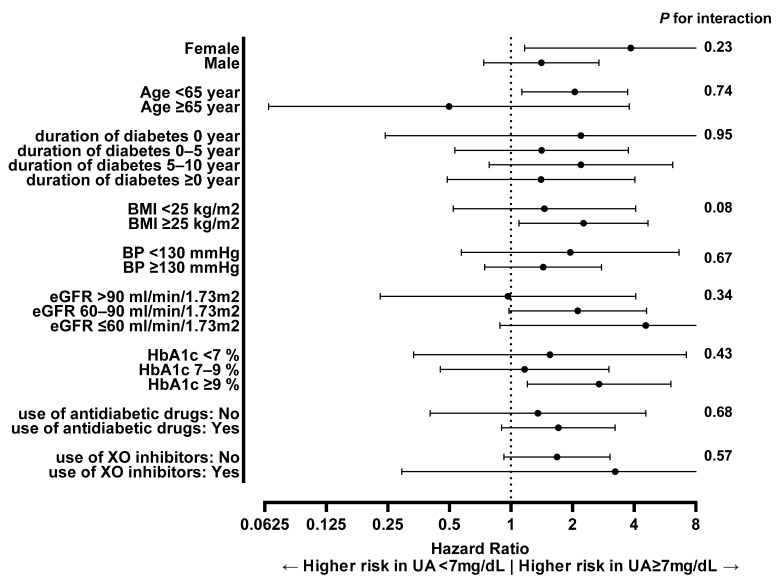
Subgroup analysis of the association between hyperuricemia and the progression of retinopathy in the SDR cohort. Forest plot displaying the hazard ratios (black circles) and 95% confidence intervals (horizontal lines) for retinopathy development in the SUA ≥ 7 mg/dL (416 μmol/L) group versus the SUA < 7 mg/dL group. The data were stratified according to key demographic and clinical variables. The *p*-values for the interaction are shown in the right column.

To assess the consistency of the impact of hyperuricemia on retinopathy progression in the SDR cohort, subgroup analyses were performed across various clinical and demographic strata (Figure 6). In contrast to the findings in the NDR cohort, where a significant interaction with HbA1c levels was observed, the association between hyperuricemia (SUA ≥ 7.0 mg/dL) and an increased risk of DR progression in the SDR cohort was generally homogenous across all analyzed subgroups, and none of the stratified variables demonstrated a statistically significant interaction (all *p* for interaction > 0.05). Notably, the *p* value for the interaction regarding HbA1c was 0.43, indicating that the detrimental effect of elevated uric levels on retinopathy progression in this more advanced stage is robust and independent of baseline glycemic control. However, within these sub-populations, hyperuricemia was significantly associated with DR progression, particularly among patients who were female, younger (age < 65 years), obese (BMI ≥ 25 kg/m^2^), and those with poorly controlled blood glucose (HbA1c ≥ 9%), as their respective 95% confidence intervals for the hazard ratios were entirely above the 1.0 threshold (Figure 6). These results suggest that while hyperuricemia acts as a consistent predictor across diverse patient profiles in the SDR stage, its adverse impact may be especially unmasked in specific high-risk subpopulations.

**Figure 7:**
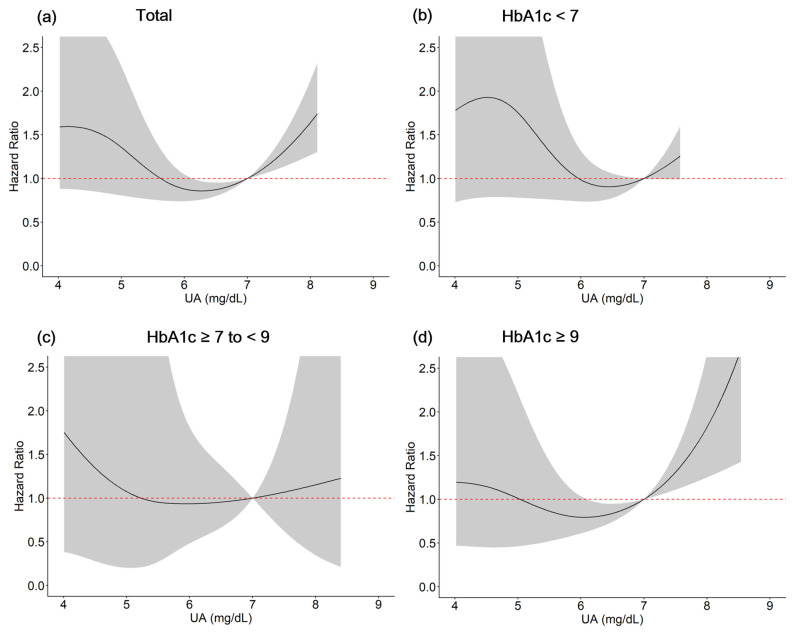
Dose–response relationship between SUA levels and the risk of retinopathy progression in the SDR cohort. Restricted cubic spline (RCS) curves show the hazard ratios (solid black lines) and 95% confidence intervals (shaded areas) for the progression of diabetic retinopathy in the SDR cohort. The reference SUA level was set at 7.0 mg/dL (horizontal red dashed line at hazard ratio = 1.0). All hazard ratios are presented relative to this reference concentration. Curves are presented for (**a**) the total SDR cohort, and stratified by baseline HbA1c levels: (**b**) HbA1c < 7%, (**c**) HbA1c 7% to <9%, and (**d**) HbA1c ≥ 9%.

To determine whether this relationship persisted in more advanced stages of the disease, we performed a similar restricted cubic spline (RCS) analysis in the SDR cohort (Figure 7). In the total SDR cohort, the risk of retinopathy progression demonstrated a continuous increase, and the hazard ratio clearly exceeded 1.0 as SUA levels rose above the 7.0 mg/dL reference value (Figure 7a). This upward trend in risk above 7.0 mg/dL was consistently observed across all stratified subgroups (Figure 7b–d). Notably, this dose–response relationship was most pronounced and accelerated in the subgroup with severe hyperglycemia (HbA1c ≥ 9%), where the hazard ratio for progression increased sharply as SUA levels increased (Figure 7d). Moreover, consistent with the NDR cohort, SUA levels between 6.0 and 7.0 mg/dL tended to be associated with hazard ratios < 1.0, reflecting a comparatively lower risk of progression. These findings robustly demonstrate that while elevated SUA levels above 7.0 mg/dL consistently correlate with a high risk of retinopathy progression in the SDR cohort, this longitudinal association is further amplified under the conditions of concurrent severe hyperglycemia.

### 3.5. Multivariable Analyses and Propensity Score Matched Analyses

The results of the Cox proportional hazards analysis for the NDR cohort are presented in Table 3. The assessment of Schoenfeld residuals confirmed that the proportional hazards assumption was satisfied for all individual variables and the overall models (all individual and GLOBAL *p* > 0.05).

In the univariable analysis, hyperuricemia (SUA ≥ 7 mg/dL) was not significantly associated with DR onset (HR 1.08, 95% CI [0.79–1.48], *p* = 0.65). Similarly, in the fully adjusted multivariable model for the entire cohort (total model), hyperuricemia was not associated with DR onset (HR 1.26, 95% CI [0.90–1.77], *p* = 0.18). In this comprehensive model, age ≥ 65 years (HR 1.35, 95% CI [1.00–1.82], *p* = 0.049), higher HbA1c (HR 1.12, 95% CI [1.06–1.17], *p* < 0.00001), higher integrated eGFR (HR 1.01, 95% CI [1.00–1.01], *p* = 0.02), and the use of antidiabetic drugs (HR 1.41, 95% CI [1.12–1.78], *p* = 0.003) were significant predictors of DR onset.

In the multivariable analyses stratified by glycemic status, the impact of hyperuricemia varied substantially across models. Hyperuricemia was not significantly associated with the outcome in Model 1 (HbA1c < 7%) or Model 2 (HbA1c 7% to < 9%). However, in Model 3 (HbA1c ≥ 9%), hyperuricemia was significantly and independently associated with DR onset, showing more than a two-fold increase in risk (HR 2.42, 95% CI [1.40–4.18], *p* = 0.002).

Table 4 displays the results of the Cox proportional hazards analysis for the SDR cohort. In the preliminary univariable analysis, age ≥ 65 years violated the proportional hazards assumption (Schoenfeld residual *p* = 0.03). To rigorously address this violation, age was incorporated as a stratifying variable (strata) in all the subsequent multivariable models. This stratification successfully stabilized the models, with all other covariates and the GLOBAL model tests satisfying the proportional hazards assumption (GLOBAL *p* ≥ 0.49 across all models).

In the univariable analysis, hyperuricemia showed a trend toward an increased risk but did not reach statistical significance (HR 1.69, 95% CI [0.96–2.96], *p* = 0.07). However, in the multivariable model adjusted for the entire SDR cohort (total model), hyperuricemia was significantly and independently associated with retinal progression, doubling the hazard ratio (HR 2.03, 95% CI [1.13–3.69], *p* = 0.02).

In the multivariable analyses stratified by glycemic status, hyperuricemia did not show significant associations in Model 1 (HbA1c < 7%) or Model 2 (HbA1c 7% to <9%), where the confidence intervals for several covariates (such as XO inhibitors) diverged due to the small number of events or sample sizes within these specific subgroups. In contrast, in Model 3 (HbA1c ≥ 9%), hyperuricemia demonstrated a remarkably high and independent risk for retinal progression, with nearly a four-fold increase in hazard (HR 3.77, 95% CI [1.52–9.39], *p* = 0.004). Male sex was also a strong independent predictor in this subgroup (HR 4.08, 95% CI [1.16–14.33], *p* = 0.03).

**Figure 8:**
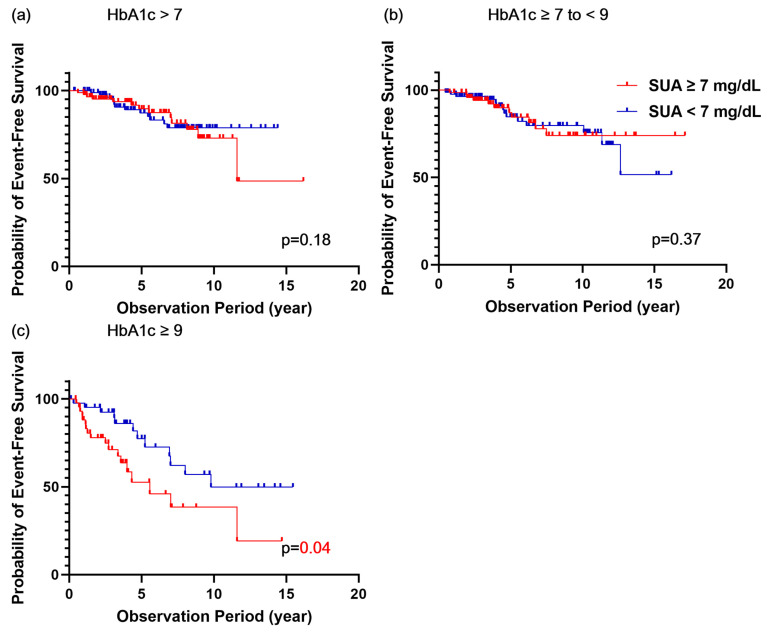
Kaplan–Meier survival analysis for DR onset after propensity score matching. (**a**) Matched cohort with HbA1c < 7%. (**b**) Matched cohort with 7% ≤ HbA1c < 9%. (**c**) Matched cohort with HbA1c ≥ 9%. A serum urate level of 7 mg/dL is equivalent to 416 μmol/L.

To further validate this finding, we performed propensity score matching for each HbA1c stratum. After matching, baseline covariates were mostly well-balanced (SMD < 0.1 for most variables, except for findings with a low frequency of positivity) (Appendix A: Table A1, Table A2 and Table A3). A subsequent Kaplan–Meier analysis of the matched cohorts confirmed the previous findings: hyperuricemia was significantly associated with a higher incidence of DR only in the group with HbA1c ≥ 9% (*p* = 0.04) (Figure 8a–c).

## 4. Discussion

The principal finding of this longitudinal study was that hyperuricemia is independently associated with the onset of diabetic retinopathy, specifically in patients with poor glycemic control (HbA1c ≥ 9%). Similarly, DR progression from SDR was associated with hyperuricemia in patients with HbA1c ≥ 9%.

The primary factor explaining why our unstratified overall analysis did not replicate the linear risk increment observed in the Tenri cohort [17] likely resides in the stark difference in baseline glycemic control. Our study population presented with a notably worse baseline HbA1c (8.23%) than Tenri cohort does (7.25%). In such a poorly controlled cohort, glucose-induced uricosuria is widely active [18]; thus, this potent physiological masking effect can wash out the underlying clinical association of elevated SUA in the overall population. By taking this glycemic interference into account, a distinct clinical characteristic of hyperuricemia under marked hyperglycemia was revealed by capturing a J-shaped dose–response relationship between SUA and DR exclusively within the hyperglycemia (HbA1c ≥ 9%) stratum. To provide a mechanistic hypothesis to explain these stratified findings, particular attention must be paid to the distinct vulnerability of the retina to oxidative stress, especially under hyperglycemic conditions. This susceptibility is hypothesized to stem from the high metabolic activity and abundant mitochondrial density inherent to retinal tissues [19]. Xanthine oxidase (XO), the rate-limiting enzyme in UA production, is a major source of reactive oxygen species (ROS) that contributes to vascular inflammation [20]. The potential upregulation of XO activity induced by oxidative stress [21] through post-translational modification converting xanthine dehydrogenase to the XO form, particularly when localized within the retinal tissue [22], may exert an excessive pro-oxidant burden in the presence of severe hyperglycemia. On the other hand, if the direct cellular toxicity of SUA itself or its pro-oxidant effects inside endothelial cells via URAT1 influx [23] were the primary driver, the risk of retinopathy should scale linearly with absolute SUA levels regardless of glycemic control; however, this was not the case with our data. By stratifying the cohort by glycemic status, we may isolate a specific subpopulation under severe hyperglycemia who maintain elevated SUA levels despite the suppression induced by glucose-mediated uricosuria [18].

Established risk factors for the onset of DR include age, blood pressure [24], sex, eGFR [25], and BMI [26]. Hyperuricemia and DR share risk factors, including advanced age, hypertension, impaired renal function, and elevated BMI [27,28]. While hyperuricemia was independently associated with DR in patients with high HbA1c levels in this study, it is important to acknowledge that these clinical features of type 2 diabetes frequently cluster with hyperuricemia, suggesting a potential synergistic aggregation of metabolic risk factors. Furthermore, in both cohorts, a higher continuous eGFR was significantly associated with the onset and progression of DR. This finding likely reflects the phenomenon of early diabetic glomerular hyperfiltration, a phase characterized by increased systemic and retinal microvascular hemodynamic shear stress, which concurrently predisposes the retinal vasculature to early endothelial damage.

Relative to the NDR cohort, hyperuricemia in the overall SDR cohort is independently associated with DR progression after multivariable adjustment, demonstrating its occult association once the initial microvascular injury is established. This detrimental association was particularly pronounced in female, younger (<65 years), and obese (BMI ≥ 25 kg/m^2^) patients. Biologically, clinical hyperuricemia in females may reflect a profound clustering of metabolic syndromes that override natural estrogenic protection [29]. In younger individuals, who are less burdened by age-related vascular damage [30], inflammation and microvascular injury in the retina associated with SUA may be more pronounced. In obese patients, intracellular UA potentially triggers oxidative stress [31]. Hyperuricemia indicates an underlying state of clinical obesity rather than simple obesity [32], which is characterized by chronic inflammation that may contribute to the risk of retinopathy in obese individuals. With respect to sex, independent of the clinical significance of hyperuricemia, attention must be paid to the increased HR of DR progression in males under hyperglycemic conditions. A recent report suggested that DR may manifest differently by sex, with women potentially at higher risk of developing macular edema, as opposed to men who may be at greater risk of PDR [33]. However, these findings require cautious interpretation; the formal tests for interaction were not statistically significant, likely due to the limited statistical power from the small SDR cohort size (N = 270; hyperuricemia n = 37). Thus, these stratified results do not definitively prove statistical effect modification but rather highlight specific vulnerable subpopulations wherein the adverse risk associated with elevated SUA is most readily unmasked.

Although clinical reports on the influence of SUA-lowering drugs on the risk of DR onset are lacking, animal studies [34] have shown that the administration of allopurinol improves the b-wave in electroretinography (ERG) in diabetic rats. However, because of the non-interventional observational design of our study, XO inhibitors were likely used selectively in patients with hyperuricemia. At the very least, no clear evidence has been obtained to actively support its inhibitory effects on the development or progression of DR. Due to the extremely low frequency of uricosuric drug use, it is difficult to draw a definitive conclusion regarding its association with the development or progression of retinopathy in the present study. Further investigations of a larger sample size, considering the treatment duration and dosage, are warranted. Among antidiabetic drugs, SGLT2 inhibitors lower blood glucose levels by promoting urinary glucose excretion, and like hyperglycemia-induced glycosuria, they also have the potential to lower SUA levels by inhibiting its reabsorption [35]. However, because the observation period of this study dates far back, the number of patients using SGLT2 inhibitors was extremely small. Currently, SGLT2 inhibitors are actively prescribed to patients with renal impairment [36], which could potentially influence the findings of this study by ameliorating the retinal damage [37].

From a clinical standpoint, these findings carry important implications for personalized risk stratification. Specifically, routine measurement of serum uric acid may offer substantial incremental prognostic value when integrated into existing clinical prediction models for diabetic retinopathy, particularly for patients presenting with poorly controlled glycemia (HbA1c ≥ 9.0%). Identifying hyperuricemia within this vulnerable subpopulation could signal the need for more intensive screening schedules and aggressive comprehensive metabolic management. Ultimately, well-designed prospective interventional trials are highly warranted to determine whether targeted uric acid-lowering therapy can effectively prevent the initiation or delay the progression of diabetic microvascular injuries.

Some limitations should be acknowledged. Principally, its observational design precludes definitively establishing a causal relationship between hyperuricemia and DR, and the potential for residual confounding cannot be entirely ruled out. Furthermore, because multiple subgroup and interaction analyses were performed across distinct metabolic strata, the possibility of chance findings arising from multiple comparisons should be considered. Additionally, although renal function was adjusted for using baseline eGFR in our multivariable models, renal impairment, hyperuricemia, and diabetic microvascular complications are intimately intertwined pathogenetically. Consequently, the presence of residual renal-related confounding remains a distinct statistical limitation. Second, our data derive from a single specialized diabetes center in Japan, raising the possibility that treatment variations at other centers could yield different results. Finally, given that hyperuricemia treatment guidelines vary across ethnicities [8] and pharmacological treatment for asymptomatic hyperuricemia (hyperuricemia without gout) is unique to Japan, caution is warranted when extrapolating our findings beyond Japanese populations. However, while routine treatment for hyperuricemia is not established in most countries, our findings are meaningful in serving as a reminder that diabetic microvascular complications should be taken into account during the measurement and interpretation of SUA levels.

## 5. Conclusions

The coexistence of hyperuricemia was independently associated with the onset and progression of DR in patients with T2D and poor glycemic control. Hyperuricemia may be associated with an increased risk for the development of retinopathy in this high-risk population.

## Figures and Tables

**Table 1:** Baseline characteristics of patients with NDR (N = 1702).

	Total N = 1702	UA ≥ 7 mg/dL N = 220	UA < 7 mg/dL N = 1482	*p*-Value
Male/Female N (%)	1358 (79.8)/344 (20.2)	199 (90.45)/21 (9.55)	1159 (78.2)/323 (21.8)	<0.0001
Age (years)	54.78 ± 10.88	52.97 ± 11.01	55.05 ± 10.83	0.008
Duration of diabetes (years) N (%)				0.25
0	178 (10.5)	18 (8.18)	160 (10.8)	
>0, <5	933 (54.8)	131 (59.55)	802 (54.12)	
≥5, <10	306 (18)	32 (14.55)	274 (18.49)	
≥10	285 (16.7)	39 (17.73)	246 (16.6)	
BMI (kg/m^2^)	25.58 ± 4.3	27.67 ± 4.87	25.27 ± 4.12	<0.0001
BMI (≥25) N (%)	861 (50.9)	149 (68.04)	712 (48.3)	<0.0001
HbA1c (%)	8.23 ± 2.01	7.8 ± 1.81	8.3 ± 2.03	0.0006
HbA1c N (%)				0.0003
<7	536 (31.5)	95 (43.18)	441 (29.76)	
≥7, <9	694 (40.8)	78 (35.45)	616 (41.57)	
≥9	472 (27.7)	47 (21.36)	425 (28.68)	
BP (≥130/80 mmHg) N (%)	963 (57.8)	146 (68.22)	817 (56.23)	0.0009
BP (≥140/90 mmHg) N (%)	493 (29.6)	80 (37.38)	413 (28.42)	0.007
eGFR (mL/min/1.73 m^2^)	83.49 ± 19.34	76.43 ± 17.79	84.53 ± 19.35	<0.0001
eGFR N (%)				<0.0001
≥90	519 (31.1)	470 (32.4)	49 (23)	
<90, ≥60	1032 (62.0)	901 (62.1)	131 (61.5)	
<60	113 (6.7)	80 (5.5)	33 (15.5)	
UA (mg/dL)	5.48 ± 1.34	7.71 ± 0.74	5.15 ± 1.06	NA
Creatinine (mg/dL)	0.76 ± 0.18	0.85 ± 0.2	0.74 ± 0.17	<0.0001
Antidiabetic drugs N (%)	851 (50)	97 (44.09)	754 (50.88)	0.0003
SGLT2 inhibitors N (%)	37 (2.17)	4 (1.82)	33 (2.23)	0.69
Xanthine Oxidase Inhibitors N (%)	53 (3.1)	24 (10.91)	29 (1.96)	<0.0001
Uricosuric medications N (%)	13 (0.8)	6 (2.73)	7 (0.47)	0.0003

A duration of 0 years refers to patients who were newly diagnosed at the initial visit. NA: not applicable.

**Table 2:** Baseline characteristics of patients with SDR (N = 270).

	Total N = 270	UA ≥ 7 mg/dL N = 37	UA < 7 mg/dL N = 233	*p*-Value
Male/Female N (%)	206 (76.3)/64 (23.7)	29 (78.38)/8 (21.62)	177 (75.97)/56 (24.03)	0.84
Age (years)	57.85 ± 10.61	56.51 ± 11.88	58.06 ± 10.41	0.41
Duration of diabetes (years) N (%)				0.94
0	26 (9.63)	3 (8.11)	23 (9.87)	
>0, <5	85 (31.48)	12 (32.43)	73 (31.33)	
≥5, <10	50 (18.52)	8 (21.62)	42 (18.03)	
≥10	109 (40.37)	14 (37.84)	95 (40.77)	
BMI (kg/m^2^)	25.31 ± 4.44	27.37 ± 4.31	24.98 ± 4.38	0.003
BMI (≥25) N (%)	124 (46.79)	26 (72.22)	98 (42.79)	0.001
HbA1c (%)	8.37 ± 1.73	8.33 ± 1.86	8.38 ± 1.71	0.86
HbA1c N (%)				0.92
<7	61 (22.59)	9 (24.32)	52 (22.32)	
≥7, <9	126 (46.67)	16 (43.24)	110 (47.21)	
≥9	83 (30.74)	12 (32.43)	71 (30.47)	
BP (≥130/80 mmHg) N (%)	181 (68.05)	29 (80.56)	152 (66.09)	0.09
BP (≥140/90 mmHg) N (%)	111 (41.73)	19 (52.78)	92 (40)	0.20
eGFR (mL/min/1.73 m^2^)	81.58 ± 22.09	66.71 ± 26.52	84.49 ± 19.96	<0.0001
eGFR N (%)				<0.0001
≥90	82 (30.9)	76 (33.3)	6 (16.2)	
<90, ≥60	148 (55.8)	131 (57.5)	17 (45.9)	
<60	35 (13.2)	21 (9.2)	14 (37.8)	
SUA (mg/dL)	5.53 ± 1.44	7.95 ± 1.01	5.15 ± 1.07	NA
Creatinine (mg/dL)	0.78 ± 0.26	1.00 ± 0.39	0.75 ± 0.23	<0.0001
Antidiabetic drugs N (%)	160 (59.26)	26 (70.27)	134 (57.51)	0.15
Xanthine oxidase inhibitors N (%)	15 (5.56)	6 (16.22)	9 (3.86)	0.009
Uricosuric medications N (%)	2 (0.74)	1 (2.7)	1 (0.43)	0.26

A duration of 0 years refers to patients who were newly diagnosed at the initial visit. NA: not applicable.

**Table 3:** Univariable and multivariable Cox proportional hazards analysis for DR onset in the NDR cohort.

Variables	Statistics	Univariable Analysis	Multivariable: Total [Events: 363]	Multivariable: Model 1 (HbA1c < 7%) [Events: 68]	Multivariable: Model 2 (HbA1c ≥ 7, <9) [Events: 149]	Multivariable: Model 3 (HbA1c ≥ 9) [Events: 146]
SUA (≥7/<7)	HR [95% CI]	1.08 [0.79–1.48]	1.26 [0.90–1.77]	1.51 [0.81–2.83]	0.65 [0.34–1.25]	2.42 [1.40–4.18]
	Cox *p*-value	*p* = 0.65	*p* = 0.18	*p* = 0.20	*p* = 0.20	***p* = 0.002**
	Schoenfeld *p*	*p* = 0.50	*p* = 0.69	*p* = 0.40	*p* = 0.63	*p* = 0.90
Sex (Male/Female)	HR [95% CI]	0.95 [0.73–1.23]	1.03 [0.78–1.34]	0.89 [0.43–1.82]	1.02 [0.69–1.50]	1.18 [0.75–1.83]
	Cox *p*-value	*p* = 0.68	*p* = 0.85	*p* = 0.75	*p* = 0.93	*p* = 0.48
	Schoenfeld *p*	*p* = 0.76	*p* = 0.86	*p* = 0.96	*p* = 0.10	*p* = 0.07
Age (A: <65/B: ≥65)	HR [95% CI]	1.15 [0.87–1.52]	1.35 [1.00–1.82]	0.76 [0.37–1.56]	1.37 [0.90–2.09]	1.87 [1.05–3.34]
	Cox *p*-value	*p* = 0.31	***p* = 0.049**	*p* = 0.46	*p* = 0.14	***p* = 0.03**
	Schoenfeld *p*	*p* = 0.55	*p* = 0.55	*p* = 0.14	*p* = 0.45	*p* = 0.09
BMI (≥25/<25)	HR [95% CI]	1.06 [0.87–1.31]	0.98 [0.80–1.22]	0.92 [0.56–1.51]	1.06 [0.76–1.47]	0.79 [0.56–1.12]
	Cox *p*-value	*p* = 0.55	*p* = 0.88	*p* = 0.74	*p* = 0.75	*p* = 0.19
	Schoenfeld *p*	*p* = 0.20	*p* = 0.23	*p* = 0.18	*p* = 0.66	*p* = 0.62
HbA1c (%)	HR [95% CI]	1.15 [1.10–1.20]	1.12 [1.06–1.17]	2.84 [1.26–6.41]	1.49 [1.11–2.01]	0.99 [0.90–1.10]
	Cox *p*-value	***p* = 0.00001**	***p* = 0.00001**	***p* = 0.01**	***p* = 0.009**	*p* = 0.88
	Schoenfeld *p*	*p* = 0.055	*p* = 0.08	*p* = 0.89	*p* = 0.32	*p* = 0.99
eGFR (mL/min/1.73 m^2^)	HR [95% CI]	1.01 [1.00–1.01]	1.01 [1.00–1.01]	0.99 [0.97–1.01]	1.00 [0.99–1.01]	1.01 [1.00–1.02]
	Cox *p*-value	***p* = 0.001**	***p* = 0.02**	*p* = 0.19	*p* = 0.85	***p* = 0.003**
	Schoenfeld *p*	*p* = 0.88	*p* = 0.97	*p* = 0.60	*p* = 0.58	*p* = 0.49
XO inhibitors (Yes/No)	HR [95% CI]	1.58 [0.93–2.70]	1.48 [0.85–2.59]	0.97 [0.33–2.88]	1.76 [0.68–4.54]	1.37 [0.53–3.52]
	Cox *p*-value	*p* = 0.09	*p* = 0.17	*p* = 0.96	*p* = 0.24	*p* = 0.51
	Schoenfeld *p*	*p* = 0.16	*p* = 0.22	*p* = 0.18	*p* = 0.28	*p* = 0.63
Antidiabetic drugs (Yes/No)	HR [95% CI]	1.73 [1.41–2.12]	1.41 [1.12–1.78]	2.13 [1.27–3.60]	1.02 [0.73–1.43]	1.29 [0.86–1.92]
	Cox *p*-value	***p* = 0.00001**	***p* = 0.003**	***p* = 0.004**	*p* = 0.91	*p* = 0.22
	Schoenfeld *p*	*p* = 0.57	*p* = 0.68	*p* = 0.64	*p* = 0.92	*p* = 0.32
GLOBAL Model Test	Schoenfeld *p*	—	***p* = 0.45**	***p* = 0.60**	***p* = 0.60**	***p* = 0.27**

HR, hazard ratio; CI, confidence interval. Bold type indicates statistically significant Cox p-value (p<0.05). A serum urate level of 7 mg/dL is equivalent to 416 µmol/L.

**Table 4:** Univariable and multivariable Cox proportional hazards regression analysis for DR progression in the SDR cohort.

Variables	Statistics	Univariable Analysis	Multivariable: Total [Events: 79]	Multivariable: Model 1 (HbA1c < 7%) [Events: 68]	Multivariable: Model 2 (HbA1c ≥ 7, <9) [ Events: 149]	Multivariable: Model 3 (HbA1c ≥ 9) [Events: 146]
SUA (≥7/<7)	HR [95% CI]	1.69 [0.96–2.96]	2.03 [1.13–3.69]	1.08 [0.21–5.46]	1.96 [0.71–5.43]	3.77 [1.52–9.39]
	Cox *p*-value	*p* = 0.07	***p* = 0.02**	*p* = 0.93	*p* = 0.20	***p* = 0.004**
	Schoenfeld *p*	*p* = 0.47	*p* = 0.59	*p* = 0.09	*p* = 0.54	*p* = 0.33
Sex (Male/Female)	HR [95% CI]	1.71 [0.96–3.04]	1.65 [0.92–2.95]	1.42 [0.23–8.66]	1.34 [0.59–3.04]	4.08 [1.16–14.33]
	Cox *p*-value	*p* = 0.07	*p* = 0.09	*p* = 0.70	*p* = 0.48	***p* = 0.03**
	Schoenfeld *p*	*p* = 0.12	*p* = 0.11	*p* = 0.31	*p* = 0.68	*p* = 0.11
Age (A: <65/B: ≥65)	HR [95% CI]	0.60 [0.34–1.03]	(Stratified)	(Stratified)	(Stratified)	(Stratified)
	Cox *p*-value	*p* = 0.06	—	—	—	—
	Schoenfeld *p*	*p* = 0.03	—	—	—	—
BMI (≥25/<25)	HR [95% CI]	0.81 [0.52–1.27]	0.69 [0.43–1.11]	1.08 [0.29–4.02]	0.77 [0.37–1.62]	0.57 [0.27–1.18]
	Cox *p*-value	*p* = 0.35	*p* = 0.13	*p* = 0.91	*p* = 0.49	*p* = 0.13
	Schoenfeld *p*	*p* = 0.34	*p* = 0.61	*p* = 0.80	*p* = 0.95	*p* = 0.31
HbA1c (%)	HR [95% CI]	1.12 [1.01–1.25]	1.05 [0.93–1.19]	0.63 [0.12–3.24]	1.71 [0.82–3.53]	0.77 [0.57–1.04]
	Cox *p*-value	***p* = 0.03**	*p* = 0.40	*p* = 0.58	*p* = 0.15	*p* = 0.08
	Schoenfeld *p*	*p* = 0.83	*p* = 0.74	*p* = 0.30	*p* = 0.072	*p* = 0.49
eGFR (mL/min/1.73 m^2^)	HR [95% CI]	1.01 [1.00–1.02]	1.01 [1.00–1.02]	1.00 [0.96–1.05]	1.02 [1.00–1.04]	1.00 [0.98–1.01]
	Cox *p*-value	*p* = 0.13	*p* = 0.28	*p* = 0.84	***p* = 0.03**	*p* = 0.59
	Schoenfeld *p*	*p* = 0.85	*p* = 0.46	*p* = 0.48	*p* = 0.73	*p* = 0.94
XO inhibitors (Yes/No)	HR [95% CI]	0.79 [0.25–2.51]	0.85 [0.26–2.78]	†	†	1.27 [0.36–4.51]
	Cox *p*-value	*p* = 0.69	*p* = 0.79	*p* = 0.999	*p* = 0.998	*p* = 0.71
	Schoenfeld *p*	*p* = 0.64	*p* = 0.76	*p* = 1.00	*p* = 1.00	*p* = 0.95
Antidiabetic drugs (Yes/No)	HR [95% CI]	1.67 [1.04–2.70]	1.36 [0.80–2.32]	2.29 [0.63–8.27]	0.73 [0.35–1.52]	2.60 [0.75–8.95]
	Cox *p*-value	***p* = 0.04**	*p* = 0.25	*p* = 0.21	*p* = 0.40	*p* = 0.13
	Schoenfeld *p*	*p* = 0.98	*p* = 0.87	*p* = 0.36	*p* = 0.64	*p* = 0.70
GLOBAL Model Test	Schoenfeld *p*	—	***p* = 0.81**	***p* = 0.49**	***p* = 0.66**	***p* = 0.53**

Age (A: <65/B: ≥65) is handled as a stratifying variable in the multivariable models to satisfy the proportional hazards assumption.HR, hazard ratio; CI, confidence interval. Bold type indicates statistically significant Cox p-value (p<0.05). † Confidence intervals diverged due to the small number of events or sample size within this subgroup.

## Data Availability

The data supporting the findings of this study are available from the corresponding author upon reasonable request and subject to prior approval from the data-contributing institution.

## Abbreviations

The following abbreviations are used in this manuscript:

Abbreviation	Full Term
ANOVA	Analysis of Variance
BMI	Body Mass Index
BP	Blood Pressure
CI	Confidence Interval
DR	Diabetic Retinopathy
ECM	Extracellular Matrix
eGFR	Estimated Glomerular Filtration Rate
ER	Endoplasmic Reticulum
ERG	Electroretinography
GLUT9	Glucose Transporter 9
HbA1c	Glycated Hemoglobin
HR	Hazard Ratio
hs-CRP	High-Sensitivity C-Reactive Protein
IQR	Interquartile Range
NDR	No Diabetic Retinopathy
PDR	Proliferative Diabetic Retinopathy
PPDR	Pre-proliferative Diabetic Retinopathy
ROS	Reactive Oxygen Species
SD	Standard Deviation
SDR	Simple Diabetic Retinopathy
SGLT2	Sodium-Glucose Cotransporter 2
SMD	Standardized Mean Difference
SUA	Serum Uric Acid
T2D	Type 2 Diabetes Mellitus
UA	Uric Acid
URAT1	Urate Transporter 1
VEGF	Vascular Endothelial Growth Factor
XO	Xanthine Oxidase

## Appendix A

### Appendix A.1

**Table A1:** Baseline characteristics of NDR patients with baseline HbA1c < 7% after propensity score matching.

NDR, HbA1c < 7	Total N = 168	SUA ≥ 7 mg/dL N = 84	SUA < 7 mg/dL N = 84	*p*-Value	SMD
Male/Female N (%)	161 (95.8)/7 (4.2)	81 (96.4)/3 (3.6)	80 (95.2)/4 (4.8)	1.00	0.06
Age (years)	54.62 ± 9.9	54.65 ± 9.73	54.58 ± 10.13	0.96	0.01
Duration of diabetes (years) N (%)				0.98	
0	14 (8.3)	7 (8.3)	7 (8.3)		0.00
>0, <5	102 (60.7)	50 (59.5)	52 (61.9)		0.05
≥5, <10	28 (16.7)	14 (16.7)	14 (16.7)		0.00
≥10	24 (14.3)	13 (15.5)	11 (13.1)		0.07
BMI (kg/m^2^)	26.39 ± 3.67	26.48 ± 3.85	26.3 ± 3.51	0.75	0.00
BMI (≥25) N (%)	100 (59.5)	52 (61.9)	48 (57.1)	0.53	0.10
HbA1c (%)	6.43 ± 0.37	6.43 ± 0.38	6.42 ± 0.37	0.85	0.27
BP (≥130/80 mmHg) N (%)	110 (65.5)	53 (63.1)	57 (67.9)	0.52	0.10
BP (≥140/90 mmHg) N (%)	47 (28)	22 (26.2)	25 (29.8)	0.61	0.08
eGFR (mL/min/1.73 m^2^)	75.05 ± 14.66	74.52 ± 14.48	75.58 ± 14.9	0.64	0.00
SUA (mg/dL)	6.64 ± 1.38	5.57 ± 1.03	7.7 ± 0.68	NA	2.87
Creatinine (mg/dL)	0.86 ± 0.18	0.87 ± 0.19	0.85 ± 0.16	0.56	0.29
Antidiabetic drugs N (%)	14 (8.3)	7 (8.3)	7 (8.3)	1.00	0.00
Xanthine oxidase inhibitors N (%)	2 (1.2)	0 (0.0)	2 (2.4)	0.50	0.22
Uricosuric medications N (%)	32 (19)	13 (15.5)	19 (22.6)	0.24	0.18

NA: Not Applicable.

### Appendix A.2

**Table A2:** Baseline characteristics of NDR patients with baseline HbA1c ≥ 7, <9% after propensity score matching.

NDR, HbA1c ≥ 7, <9	Total N = 138	SUA ≥ 7 mg/dL N = 69	SUA < 7 mg/dL N = 69	*p*-Value	SMD
Male/Female N (%)	115 (83.3)/23 (16.7)	58 (84.1)/11 (15.9)	57 (15.9)/12 (17.4)	0.82	0.04
Age (years)	52.46 ± 11.42	53.57 ± 11.09	51.35 ± 11.71	0.26	0.02
Duration of diabetes (years) N (%)				0.91	
0	17 (12.3)	8 (11.6)	9 (13)		0.04
>0, <5	79 (57.2)	39 (56.5)	40 (58)		0.03
≥5, <10	21 (15.2)	10 (14.5)	11 (15.9)		0.04
≥10	21 (15.2)	12 (17.4)	9 (13)		0.12
BMI (kg/m^2^)	27.7 ± 4.69	27.3 ± 4.32	28.11 ± 5.04	0.31	0.04
BMI (≥25) N (%)	93 (67.4)	45 (65.2)	48 (69.6)	0.43	0.09
HbA1c (%)	7.79 ± 0.59	7.72 ± 0.62	7.85 ± 0.57	0.20	0.37
BP (≥130/80 mmHg) N (%)	87 (63)	42 (60.9)	45 (65.2)	0.60	0.09
BP (≥140/90 mmHg) N (%)	47 (34.1)	27 (39.1)	20 (29)	0.21	0.22
eGFR (mL/min/1.73 m^2^)	79.59 ± 20.13	76.96 ± 17.59	82.21 ± 22.21	0.13	0.01
SUA (mg/dL)	6.42 ± 1.37	7.56 ± 0.52	5.28 ± 0.93	NA	4.01
Creatinine (mg/dL)	0.81 ± 0.19	0.83 ± 0.17	0.8 ± 0.2	0.37	0.82
Antidiabetic drugs N (%)	8 (5.8)	4 (5.8)	4 (5.8)	1.00	0.00
Xanthine oxidase inhibitors N (%)	3 (2.2)	2 (2.9)	1 (1.4)	1.00	0.10
Uricosuric medications N (%)	66 (47.8)	33 (47.8)	33 (47.8)	0.44	0.00

NA: Not Applicable.

### Appendix A.3

**Table A3:** Baseline characteristics of NDR patients with baseline HbA1c ≥ 9% after propensity score matching.

NDR, HbA1c ≥ 9	Total N = 70	SUA ≥ 7 mg/dL N = 35	SUA < 7 mg/dL N = 35	*p*-Value	SMD
Male/Female N (%)	61 (87.1)/9 (12.9)	30 (85.7)/5 (14.3)	31 (88.6)/4 (11.4)	0.82	0.09
Age (years)	50.71 ± 10.43	50.11 ± 11.55	51.31 ± 9.31	0.26	0.01
Duration of diabetes (years) N (%)				0.91	
0	4 (5.7)	2 (5.7)	2 (5.7)		0.00
>0, <5	43 (61.4)	20 (57.1)	23 (65.7)		0.18
≥5, <10	10 (14.3)	5 (14.3)	5 (14.3)		0.00
≥10	13 (18.6)	8 (22.9)	5 (14.3)		0.22
BMI (kg/m^2^)	28.07 ± 4.37	27.82 ± 4.16	28.31 ± 4.61	0.31	0.03
BMI (25≤) N (%)	53 (75.7)	27 (77.1)	26 (74.3)	0.59	0.07
HbA1c (%)	10.68 ± 1.56	10.65 ± 1.53	10.71 ± 1.61	0.20	0.02
BP (≥130/80 mmHg) N (%)	51 (72.9)	24 (68.6)	27 (77.1)	0.60	0.19
BP (≥140/90 mmHg) N (%)	27 (38.6)	13 (37.1)	14 (40)	0.21	0.06
eGFR (mL/min/1.73 m^2^)	78.84 ± 19.64	79.7 ± 21.5	77.98 ± 17.85	0.13	0.00
SUA (mg/dL)	6.55 ± 1.42	7.67 ± 0.71	5.43 ± 1.02	NA	2.91
Creatinine (mg/dL)	0.84 ± 0.25	0.85 ± 0.28	0.84 ± 0.22	0.37	0.07
Antidiabetic drugs N (%)	6 (8.6)	3 (8.6)	3 (8.6)	1.00	0.00
Xanthine oxidase inhibitors N (%)	2 (2.9)	1 (2.9)	1 (2.9)	1.00	0.00
Uricosuric medications N (%)	48 (68.6)	25 (71.4)	23 (65.7)	1.00	0.12

NA: Not Applicable.
